# Commentary: Shared decision making for weight‐lowering medications in China

**DOI:** 10.1002/ctm2.70065

**Published:** 2024-10-24

**Authors:** Qingyi Jia, Sheyu Li

**Affiliations:** ^1^ Department of Endocrinology and Metabolism West China Hospital Sichuan University Chengdu Sichuan Province China; ^2^ Chinese Evidence‐Based Medicine Center Cochrane China Center and MAGIC China Center West China Hospital Sichuan University Chengdu Sichuan Province China

One in eight adults are suffering from obesity and its complication in 2022 worldwide, with the US leading the top prevalence of 67% adults with overweight adults. Following the western countries, China is entering a pandemic of obesity with 34.8% of adults with overweight and 14.1% with obesity and the most rapid increase of the population.^1^, ^2^ The increasing disease burden costs an estimating 2.15 million US dollars in China 2024.

NuSH agonists including GLP‐1 receptor agonists, GLP‐1/GIP dual agonists, GLP‐1/glucagon receptor dual agonists, GLP‐1/GIP/glucagon receptor triple agonists showed their efficacies in weight lowering.^3^ Recent large systematic reviews demonstrated the weight‐lowering effects of IH semaglutide in adults with overweight and obesity and trizepatide in people with type 2 diabetes.^4^, ^5^ Both received or applied their approvals to the Chinese FDA. Retatrurtide, the triple agonist, in its phase 2 trials, indicated an almost certainly 10% body weight loss adding to lifestyle modification, the efficacy might surpass any other existing weight‐lowering medications including Beinaglutide, Danuglipron, Dulaglutide, Exenatide, Loxenatide, Liraglutide, Orforglipron Mazdutide, Efinopegdutide, Surbodutide (Tirzepatide). All these medications represent competitive alternative therapies for bariatric surgery. The latest large trial for the first time demonstrated the cardiovascular benefits of semaglutide in adults with obesity but not diabetes.^4^

The plentifulness of obesity treatment medications does not mean a one‐pill‐fit‐all strategy in prescribing these medications. Generally, patients need these medications if they have difficulty in changing their lifestyles, reaching further achievements after all their efforts, or needing time‐sensitive body weight loss.

For example, osteoarthritis is a common complication of obesity and dramatically raises the risk of sport‐related injury especially when the joint bears great weight load during intensive exercise. In such cases, patients face difficulty in initiating lifestyle modification, especially the exercise. Anti‐obesity medications help prevernt such sport‐related injuries through a promising body weight loss that frees the weight load of the joint in the early phase of the obesity treatment. Gradually increasing exercise in parallel helps maintain the skeletal muscle mass and function as well as the basal metabolism rate.^6^ For people reaching their weight‐loss plateau, anti‐obesity medications may help break the balancing and allow further uptitration of physical exercise. Anaesthesia can be dangerous for some candidates of bariatric surgery and very severe obesity with impaired ventilation. Anti‐obesity medications in this case with intensive lifestyle modification may help lose 5–10% of body weight in a short period before bariatric surgery. Similar needs fit people with obesity and decompensated heart failure or other life‐threatening diseases.

Both clinicians and patients are tangling in choosing these dazzling treatment options, bringing everything back to the very beginning again—why do people lose their body weight? The motivation of obesity treatment varies across individuals. A comprehensive interview bands clinicians and patients with common and clear purposes, which allows shared decision‐making with rigorous evidence and good presentation.^7^ Shared decision‐making reflects the respect to the patients’ values and preferences and improves the compliance and the satisfaction of the healthcare receivers.^8^

**FIGURE 1:**
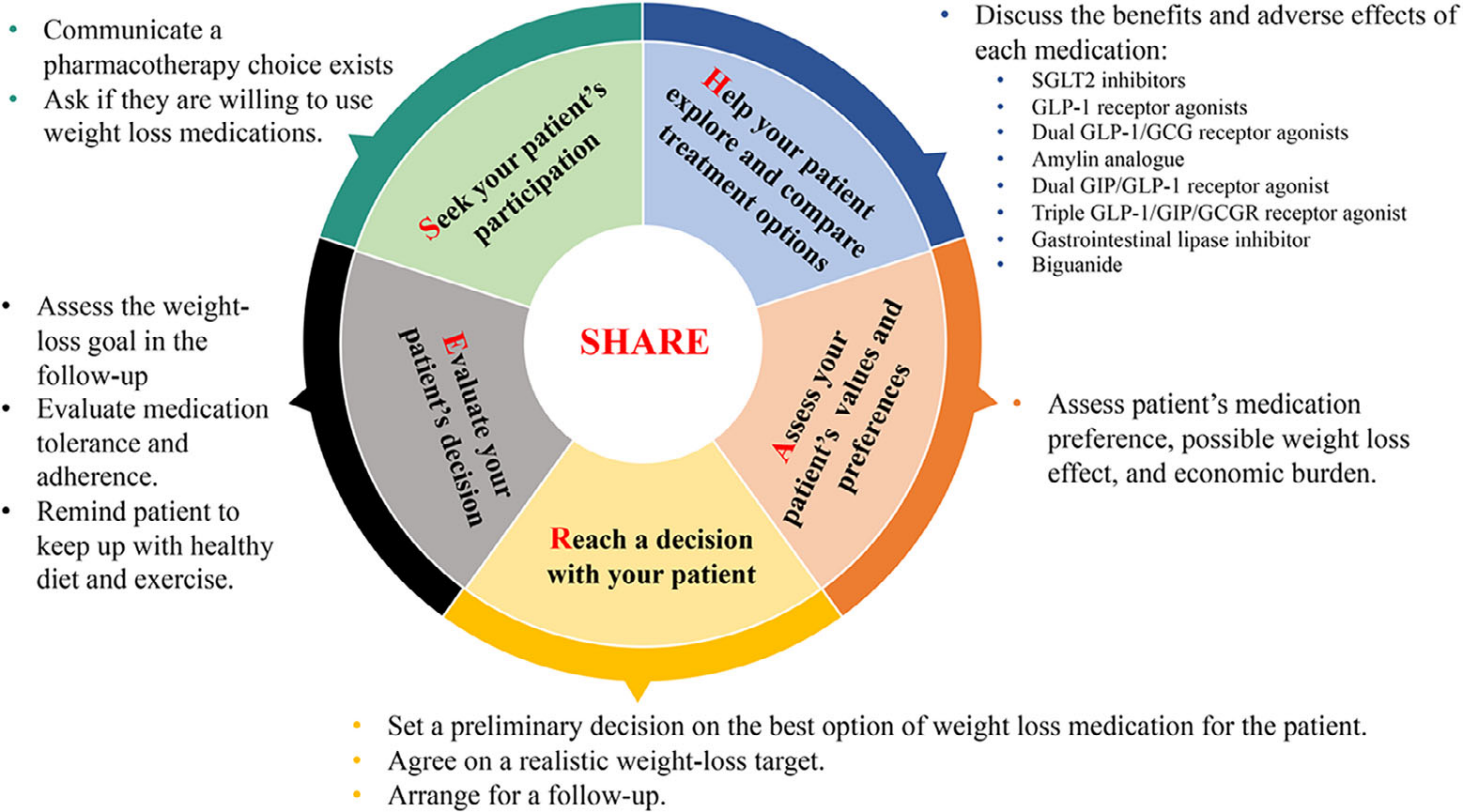
SHARE approach to decision making of pharmacotherapy for adults with obesity/overweight in China.

The SHARE approach is the most common framework for shared decision‐making including five common steps: (1) seeking for participation, (2) helping the patient with the evidence treatment options; (3) assessing the patient's values and preferences, (4) reaching a decision; and (5) evaluating the decision (Figure 1). Anchoring the individualised motivation of obesity treatment, the discussion regarding the treatment options usually involves heavy input from the patients, who may figure out the pros and cons of the treatment in their daily life. The clinicians should inspire such consideration and encourage any questions. When choosing an individual medication, all domains of GRADE Evidence to Decision (EtD) framework are relevant including sections for formulating the question, making an assessment and drawing conclusions. The latest network meta‐analysis (NMA) shows up a good example in sharing the evidence with the patients in a user‐friendly.^9^ A recent user test suggested the wide acceptability of our interactive decision aid based on our previous NMA in general practitioners. With these static and interactive digital tools, the SHARE approach fits almost all scenarios especially when the patients’ values and preferences are heterogeneous across individuals such as the choice of anti‐obesity medications. In the framework, the clinicians disseminate the evidence to the patients through the decision aids and the patients express their situation, consideration and rationales in choosing the medications. During the discussion, clinicians may find concerns and gaps of the patients’ knowledge, attitude or behaviour in managing their body shape, allowing precision medical suggestions. The full frame thus improves the adherence and satisfaction of the patients.

Among challenges, weight regain is undoubtedly the most critical concern. The Semaglutide Effects on Cardiovascular Outcomes in People with Overweight or Obesity (SELECT) trial follow‐up study, Semaglutide Treatment Effect in People with Obesity (STEP) program 1 (STEP1) showed two‐thirds weight regain of the prior weight loss after one year of withdrawal.^10^ To maintain long‐term weight‐lowering effects, most people achieving their ideal body shape have to use these medications in long term, with less than half of patients maintaining more than 5% weight loss after 52‐week withdrawal.^10^ And most of the weight loss regained in 12 years. The healthy diet and regular exercise as habits are thus critical for people trying to cease their drugs. Aiming to the physician should encourage the patient to develop a healthy diet and a regular exercise habit, while preparing the patient for the need for long‐term using this medication.

In summary, with novel medications approving their weight‐lowering effects, people with obesity expand their options when seeking to change the body shape. With heterogeneous values and preferences, motivation interview and shared decision‐making are critical for modern management of obesity and related diseases.
